# Efficacy and Safety of an Oral Low‐Dose Water‐Dispersible Turmeric Extract Capsule on Facial Skin Health in Healthy Women: A Randomized, Double‐Blind, Placebo‐Controlled Trial

**DOI:** 10.1111/jocd.70462

**Published:** 2025-09-19

**Authors:** Shefali Thanawala, Rajat Shah, Krishnaraju Venkata Alluri, Kiran Bhupathiraju, Anjali Salvi

**Affiliations:** ^1^ Nutriventia Private Limited Mumbai India; ^2^ Laila Nutraceuticals Vijayawada India; ^3^ Clinical Aesthetics and Investigative Management Service Pvt. Ltd. Mumbai India

**Keywords:** blemish, curcuminoids, facial skin health, gloss, hydration, TEWL, turmeric, WDTE60N

## Abstract

**Background:**

The role of turmeric extract (oral and topical) has been studied in the management of various skin disorders, but its potential to improve skin health in healthy individuals remains largely unexplored.

**Aim:**

To evaluate the efficacy and safety of an orally administered low‐dose, water‐dispersible turmeric extract containing 60% natural curcuminoids (WDTE60N) in reducing blemish and enhancing skin tone evenness, hydration, and gloss in healthy women.

**Methods:**

In this double‐blind, randomized, placebo‐controlled trial, women (18–40 years) with one identified facial blemish were randomized to receive WDTE60N (250 mg) or a placebo capsule once daily for 60 days. Changes in a pre‐marked blemish area on facial skin, skin hydration, transepidermal water loss (TEWL), and skin gloss from baseline to day 60 were evaluated.

**Results:**

Of 96 participants enrolled, 90 (WDTE60N, *n* = 45; placebo, *n* = 45) completed the study. The WDTE60N group showed significantly greater reductions in Antera 3D imaging readings for blemish from baseline compared to placebo starting from day 30 (*p* = 0.036) onwards to day 60 (*p* = 0.001). Significant improvement from baseline in blemish scale score on day 30 (*p* = 0.024) and day 60 (*p* = 0.001) was observed in the WDTE60N group than in the placebo group. From day 15 onward, the WDTE60N administration significantly improved skin hydration and gloss while significantly reducing TEWL (*p* = 0.001). WDTE60N was well tolerated with no major adverse events.

**Conclusion:**

WDTE60N 250 mg capsule was safe and effective in improving facial skin health in the participants. This study provides clinical evidence supporting WDTE60N as a promising cosmeceutical agent.

**Trial Registration:**

CTRI number: CTRI/2024/06/068637

## 1 Introduction

The health and radiance of facial skin are of key importance in determining skin quality and serve as a reflection of overall well‐being. Skin quality is rapidly gaining traction across the world as it significantly influences perceptions of age, attractiveness, health, and youthfulness [1, 2]. While this is relevant to both men and women, it holds particular significance for women. Particularly, unhealthy facial skin, often characterized by dryness, hyperpigmentation, and sunspots or blemishes, can notably impact self‐confidence, mood, psychological health, and even professional well‐being [3, 4]. As a result, maintaining healthy facial skin has emerged as an essential aspect of women's overall health.

Although therapies including photoprotection, topical and systemic therapies, chemical peels, and laser or light‐based therapies are available for the management of damaged or unhealthy facial skin, there is a growing demand for natural, safe, convenient, and cost‐effective alternatives, with many individuals favoring plant‐based products over prescription medications and complex procedures [5, 6, 7]. Nutraceuticals, including bioactive peptides, polysaccharides, botanical extracts, carotenoids, etc., have garnered significant attention as cosmeceuticals for maintaining skin health, owing to their nutritional benefits, safety profile, and therapeutic potential in delivering anti‐aging and skin‐reaffirming effects, supporting the concept of beauty from within [8, 9].

Turmeric root extract is one such natural ingredient with pleiotropic pharmacological actions. Interestingly, turmeric, the golden spice, has held a unique place as a readily available cosmeceutical in Indian society for centuries [10, 11]. Modern research validates this traditional use through confirmation of the potent antioxidant and anti‐inflammatory effects of its active compounds, curcuminoids, commonly known as curcumin. Research studies confirm the positive role of curcumin in several skin issues. Studies have shown that curcumin promotes wound healing by increasing angiogenesis and reducing inflammation, helps in scar reduction by inducing apoptosis of fibroblasts, combats skin aging with its potent antioxidative properties, and decreases skin pigmentation by preventing the formation of the melanin‐producing enzyme—tyrosinase and arresting the process of melanogenesis [12, 13].

Despite curcumin offering multiple health benefits, its poor absorption and low bioavailability often lead to poor compliance due to the requirement of taking multiple doses on a daily basis, especially during long‐term use. Typically, most commercially available turmeric extracts with enhanced bioavailability are formulated with a higher content of synthetic excipients and a limited content of active ingredients (6% to 20%), thus necessitating higher doses to reach meaningful systemic concentrations and elicit clinical efficacy. This highlights the need to develop an oral turmeric formulation that can provide optimum health benefits at lower doses. A low‐dose, water‐dispersible turmeric extract containing 60% natural curcuminoids (WDTE60N of Nutriventia Private Limited and Laila Nutraceuticals, India) is developed using only nature‐sourced excipients to deliver optimal benefits with a single daily low dose of 250 mg, containing 150 mg of curcuminoids. The equivalent bioavailability of curcuminoids in WDTE60N at a 10‐fold lower dose compared to the commercially available 95% turmeric extracts containing 1500 mg curcuminoids, without and with piperine, was substantiated in two human pharmacokinetic studies [14, 15].

Although the benefits of turmeric extract supplementation, either as ingested or topical formulations, have been documented for various skin diseases, its effects on promoting healthy skin—specifically in reducing blemishes and improving hydration and radiance of facial skin—remain unexplored [5]. Consequently, a gap exists in the current literature regarding the efficacy and safety of oral turmeric extract in improving the facial skin health of healthy individuals. Therefore, the present study was designed to evaluate the efficacy and safety of the WDTE60N 250 mg capsule, once a day, in reducing blemishes and enhancing various facial skin parameters, such as hydration, gloss, and skin tone evenness, in healthy adult women.

## 2 Materials and Methods

### 2.1 Study Design and Ethical Considerations

This was a single center, randomized, double‐blind, two‐arm, parallel group, prospective placebo‐controlled clinical study. The study was conducted at Clinical Aesthetics and Investigative Management Service Private Limited (CLAIMS), Mumbai, India, between 08 July 2024 and 21 October 2024. The study protocol was approved by the CLAIMS Independent Ethics Committee (Re‐Registration number: ECR/245/Indt/MH/2015/RR‐22; Approval Code: CL/172/0124/STU; Approval Date: 31/05/2024). The study protocol adhered to the ethical guidelines set forth by the Declaration of Helsinki for research involving human participants. The study was conducted in compliance with the Indian Council of Medical Research (ICMR) guidelines—National Ethical Guidelines for Biomedical and Health Research Involving Human Participants, 2017, International Conference on Harmonization‐Good Clinical Practices (ICH‐GCP) guidelines E6 (R2), NDCT RULES 2019, Declaration of Helsinki (Brazil, October 2013), and other applicable regulatory requirements. This study was registered with the Clinical Trials Registry‐India (CTRI) on 10/07/2024 (CTRI Number: CTRI/2024/06/068637). Written informed consent was provided by each participant before the initiation of the study.

### 2.2 Study Population

The eligibility criteria for study participation were as follows: women participants who (i) were aged 18–40 years, with their healthy status confirmed through medical history, physical examination, and clinical judgment of the principal investigator (PI); (ii) had a blemish on their face (such as post‐inflammatory hyperpigmentation, acne marks, age spots, sunspots) with otherwise healthy skin; (iii) were willing to discontinue or refrain from using other cosmetic products on face as well as topical and/or oral products with similar benefits or products containing sunscreen that could interfere with the study results during the course of the study; (iv) were willing to avoid intense ultraviolet (UV) exposure as far as possible for the entire study duration, comply with the study procedures, and provide informed consent. Participants were excluded from the study based on the following criteria: women (i) with significant skin damage (e.g., severe sunburn or suntan), severe acne, any cutaneous skin conditions (such as scars, moles, papules, etc.) on face, or any clinically significant systemic disease, which may interfere with study procedures or treatments; (ii) who have used collagen peptide supplements in the past 6 months, consumed high doses of vitamin C (> 500 mg/day) or any other high‐dose antioxidant product in the past month, or have been on any nutritional supplement or any therapeutic medication for any disorder for the past 45 days; (iii) with chronic gastrointestinal issues (such as frequent indigestion, gastroesophageal reflux disease) or with known allergies to any of the ingredients of the investigational products; (iv) with history of gallstones, bile duct obstructions, or bleeding disorders; (v) with a history or presence of substance abuse (alcohol, drugs, smoking); (vi) who have participated in any other experimental investigational study within the last month before the screening visit; (vii) peri‐menopausal or post‐menopausal women, pregnant women (as confirmed by a urine pregnancy test) or lactating women.

### 2.3 Study Products

*Test product*: Water‐dispersible turmeric extract capsules containing 60% natural curcuminoids (TurmXTRA60N—WDTE60N) 250 mg were manufactured by Nutriventia Private Limited, Mumbai, India, and Laila Nutraceuticals, India.

*Placebo*: Placebo capsules were manufactured as identical capsules that matched the color, size, and shape of the WDTE60N, but without the active ingredient by Nutriventia Private Limited, Mumbai, India.

### 2.4 Study Procedure

After providing written informed consent, all participants underwent screening evaluations including medical history, physical examinations, demographic and anthropometric measurements, vital sign measurement, urine pregnancy test, and blood biochemistry examinations (including complete blood count, fasting blood glucose, lipid profile, liver function tests, and renal function tests). The randomization codes were generated by the simple randomization method using the “RAND” function in Microsoft Excel. To avoid bias, the investigational products (IP) were coded and blinded prior to allocation, ensuring that the sponsor, study center, PIs, study personnel, and participants were unaware of the IP identity. The blinding and labelling were carried out by unblinded personnel at the study center. The IPs were packed in identical containers which were labeled with a randomization code. The unblinded codes were available with the PI, and blind was allowed to be broken in case of treatment emergent adverse events (AEs) or participant's safety issues.

On the baseline visit (day 0), all eligible participants were randomized to receive either 250 mg of WDTE60N or a placebo capsule. On days 0 and 30, participants were dispensed with the IP containers containing 33 capsules of either WDTE60N or placebo for 30 ± 3 days. They were instructed to take one capsule orally once daily in the morning, 30 min after breakfast, for 60 days, with water. To assess IP compliance, all participants received a diary to record daily capsule consumption. These diaries were reviewed at each visit to calculate the number of IPs used and unused. Unused IPs were collected from participants on days 30 and 60. The participant diary also included a section for recording any AEs experienced during the study period.

During the conduct of the study, use of usual toiletry products lacking skin modifying properties (soaps, shampoos, etc.) or any medical treatment not affecting the parameters of the study was allowed at the discretion of the PI. Participants were asked to abstain from any beauty treatments including but not limited to masks, facials, and exfoliating treatments at home and/or salon, fairness or bleaching creams/serums products, sauna sessions, or spa therapies that can interfere with the study treatment until study completion.

The total study period was 67 days that included 7 days of screening (Visit 1) and 60 days of treatment period (Visit 2 [baseline/day 0], Visit 3 [day 15 ± 2], Visit 4 [day 30 ± 2], and Visit 5 [day 60 ± 2]). Participants were restricted to consuming tea, coffee, or water 45 min prior to study assessments at each visit. These restrictions helped ensure that skin parameter assessments (such as Antera 3D imaging, hydration scores and transepidermal water loss [TEWL]) remained unbiased, providing a more accurate reflection of the actual status of facial skin. Before performing efficacy assessments, the test area (on the face) of each participant was cleaned, and each participant was acclimatized under controlled conditions of temperature (20°C–22°C) and humidity (40%–60%) for one hour. Changes in the pre‐marked blemish area on facial skin from baseline to the end of the study were evaluated using Antera 3D imaging (Antera software, Miravex Limited, Dublin, Ireland, Serial Number: 20205S83) as well as clinically by subjective blemish scale. The investigator assessed changes in the TEWL using VapoMeter (Delfin Technologies, Kuopio, Finland, Serial Number: SWL 4178), facial skin tone evenness via Antera 3D imaging (Antera software, Miravex Limited, Dublin, Ireland, Serial Number: 20205S83), skin hydration using MoistureMeterSC (Delfin Technologies, Kuopio, Finland, Serial Number: S/N MSC1138), and skin gloss using Skin GlossMeter (Delfin Technologies, Kuopio, Finland, Serial Number: SGM1003). Additionally, clinical photographs of the face of each participant were taken at all study visits from baseline.

### 2.5 Endpoints

The primary endpoint was to evaluate and compare the mean change in pre‐marked blemish area on facial skin using Antera 3D imaging from baseline (day 0) to the end of the treatment (day 60) between the WDTE60N and placebo groups. The secondary efficacy endpoints were to assess and compare changes from baseline between the WDTE60N and placebo groups (i) in the pre‐marked blemish areas on facial skin using Antera 3D imaging on days 15 and 30; (ii) in skin parameters including facial skin hydration, TEWL, skin gloss, and skin tone evenness at all interim study visits (days 15 and 30) until the end of the study (day 60). Additionally, subjective assessment and comparison of changes in pre‐marked blemish on facial skin using the blemish scale were conducted from baseline till day 60 between the WDTE60N and placebo groups. We subjectively evaluated the efficacy of IPs on various skin parameters (at each study visit) and in‐use tolerance (from day 15 onwards till the end of the study) using a self‐assessment questionnaire. Safety assessment of AEs was conducted by reviewing participants' diaries from day 15 onwards until day 60.

### 2.6 Study Assessment Tools

#### 2.6.1 Blemish Scale for Clinical Evaluation of Blemishes

The blemish scale is a subjective scale used to evaluate the intensity of blemish darkness by comparing it to normal skin, measured on a scale of 0–4 (Grade 0: Normal skin color without evidence of blemish; Grade 1: Barely visible blemish; Grade 2: Mildly dark blemish; Grade 3: Moderate dark blemish; Grade 4: Very dark blemish). For clinical grading of a blemish, the PI tracked a single spot over the duration of the study using this scale.

#### 2.6.2 3D Imaging Analysis for Blemish and Skin Tone Evenness

Antera 3D CS (Miravex Limited, Dublin, Ireland) is an instrument comprising an optical 3D camera used for image acquisition and analysis of the skin. It relies on the principle of multi‐directional illumination and computer‐aided reconstruction of the skin surface, illuminating the surface from different angles and using the differences between these images to reconstruct the surface in three dimensions. The skin topography and the chromophore concentration were derived from the spatial and spectral analysis of the acquired image data, obtained by illuminating the skin with light‐emitting diodes of different wavelengths (ranging from 455 to 625 nm) shining from different directions. The surface reconstructed in this way was then used for quantitative skin analysis [16]. A decrease in the 3D Antera reading (arbitrary units [au]) for skin blemish indicates a reduction in blemish on the skin. For calculating skin tone evenness, the L* (luminescence) value on a blemish as well as on neat skin was measured. Then the difference in values was calculated by subtracting the L* value on the spot from the L* value on neat skin. A decrease in the L* difference over the study duration indicates an improvement in the skin tone evenness.

#### 2.6.3 MoistureMeterSC for Evaluation of Skin Hydration

The MoistureMeterSC Compact (Delfin Technologies Ltd., Kuopio, Finland) is an instrument that measures the electrical capacitance of the skin layers, which is proportional to the water content of the surface layer of the skin. The measurement depth of the MoistureMeterSC varies and is determined by the thickness of the stratum corneum's dry layer [17, 18]. Triplicate measurements were taken, and the average value was used to minimize measurement variability. The higher reading indicates the higher moisture content of the skin (MoistureMeterSC reading < 20 indicates dry skin, 20–40 indicates normal skin, and > 40 indicates well‐hydrated skin).

#### 2.6.4 Transepidermal Water Loss by VapoMeter

Changes in the skin barrier integrity were assessed by measuring TEWL using VapoMeter (Delfin Technologies Ltd., Kuopio, Finland). The VapoMeter is equipped with a closed cylindrical chamber containing sensors that detect the change in relative humidity (%) and temperature (°C) after being placed on the skin surface. The TEWL is then measured according to the change in percentage in relative humidity [19]. To minimize measurement variability, triplicate readings were recorded, and the average value was calculated. A reduction in the VapoMeter readings indicates a decrease in TEWL from the skin and suggests an improvement in the skin barrier integrity properties. No significant change in the readings indicates that the skin barrier properties remain unaffected.

#### 2.6.5 Skin Gloss by SkinGlossMeter

SkinGlossMeter (Delfin Technologies Ltd., Kuopio, Finland, Serial No: SGM1003) was used to assess the skin gloss. The instrument contains a built‐in 635 nm red semiconductor diode laser that measures the specularly reflecting light from the skin when the laser beam is directed onto the skin surface. This reflected light then passes through an internal diffractive microstructure, where the intensity of the beam is analyzed to calculate the skin's gloss value [19, 20]. Triplicate measurements were recorded, and the average value was calculated to reduce measurement variability. An increase in the glossmeter reading indicates an increase in skin gloss.

#### 2.6.6 Self‐Assessment Questionnaires

The subjective questionnaires were designed to evaluate the efficacy and in‐use tolerance of IPs based on the participants' perceptions. The efficacy questionnaire included six questions aimed at assessing participants' perceptions regarding the effect of the IPs on various skin parameters, including skin softness and smoothness, moisturization, healthy glow, skin tone evenness, the appearance of dark spots, and blemish‐free skin. At each study visit, participants rated their responses on a scale of 1 to 5 (1 = Strongly disagree; 2 = Disagree; 3 = Neither agree nor disagree; 4 = Agree; 5 = Strongly agree). The in‐use tolerance questionnaire contained eight questions to assess the discomfort experienced by the participants.

### 2.7 Statistical Analysis

The sample size was calculated using the Cohen's D formula [21]:

$$n={{\left(Z_{\alpha /2}+Z_{\beta }\right)}^{2}}^{*}2^{*}\sigma^{2}/d^{2}$$

where *Z*_*α*/2_ = the critical value of the normal distribution at *α*/2 (e.g., for a confidence level of 95%, *α* is 0.05 and the critical value is 1.96); *Z*_*β*_ = the critical value of the normal distribution at *β* (e.g., for a power of 80%; *β* is 0.2 and the critical value is 0.84); *σ* ^2^ = Population variance; *d* = the expected difference between the sample mean and the reference value.

With an effect size of at least 0.6, a sample size of 43.6 participants in each group was required to achieve a power of 80% at a significance level (alpha) of 0.05. Considering the 10% dropout rate, a sample size of approximately 48 participants per group was considered adequate for randomization in this study.

Data were analyzed using Statistical Package for Social Sciences (SPSS) software, Version 30.0 (SPSS Inc., Chicago, Ill., USA). The data of all participants who completed all the study visits were considered for per‐protocol study analysis. The normality of data was assessed using the Shapiro–Wilk test. Descriptive analysis was used to summarize continuous parameters using the mean and standard deviation (SD) and categorical parameters using frequencies and percentages. The mean change in the outcome measure from baseline to each follow‐up visit was calculated by subtracting the mean value observed at baseline from that observed at each timepoint. The comparison of mean values between baseline and each follow‐up visit within each group was performed using paired samples *t*‐test for normally distributed parameters and the Wilcoxon signed‐rank test for non‐normally distributed parameters. Comparative analysis of mean changes from baseline to each follow‐up visit between the two groups was done using an independent sample *t*‐test (for normally distributed parameters) or the Mann–Whitney *U* test (for non‐normally distributed parameters). All *p*‐values were reported based on a two‐sided significance test, and a *p* < 0.05 was considered statistically significant.

## 3 Results

### 3.1 Participant Disposition and Characteristics

**FIGURE 1:**
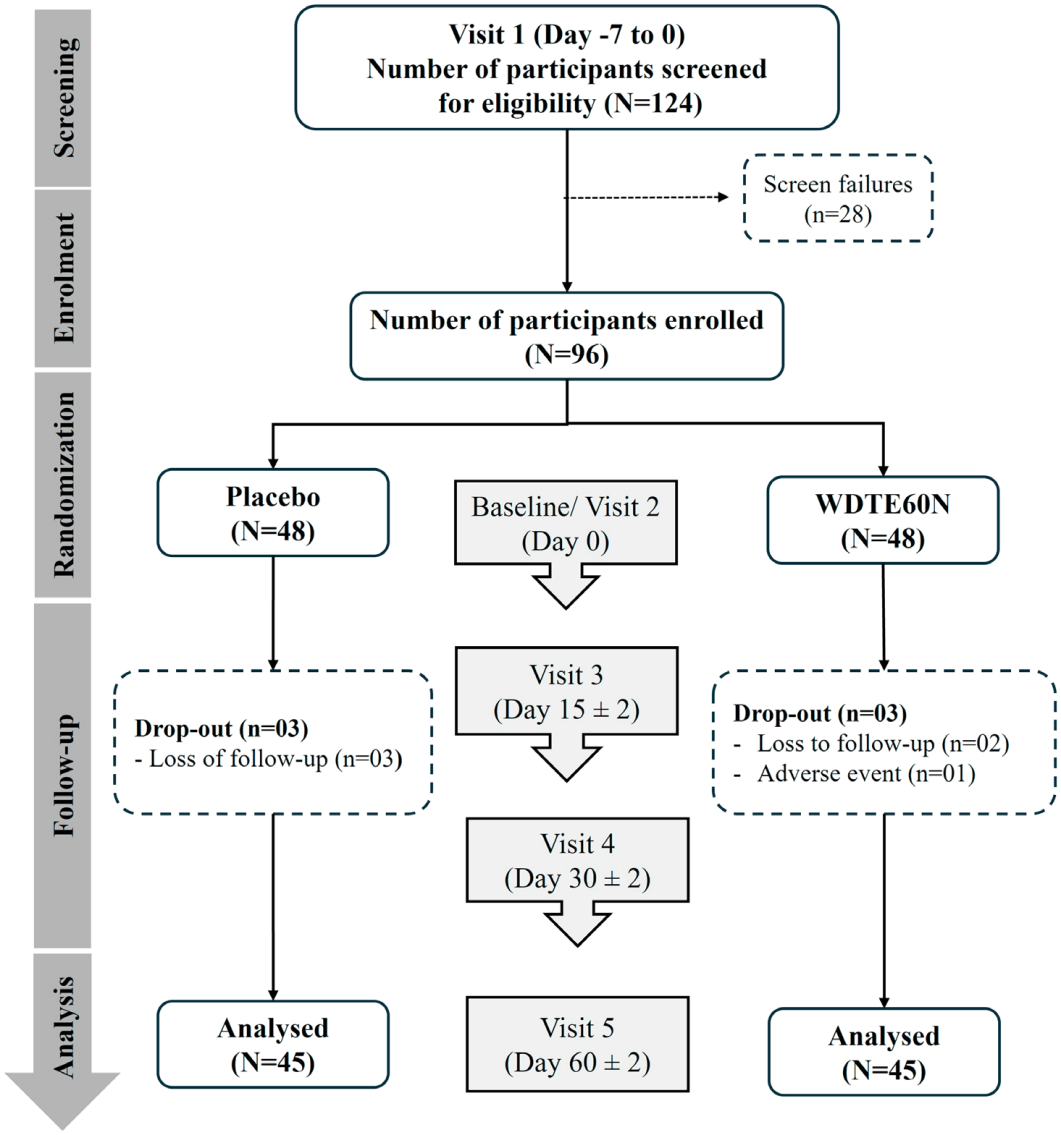
CONSORT flow diagram. WDTE60N, water‐dispersible turmeric extract containing 60% natural curcuminoids.

A total of 124 women were screened, of whom 96 were randomized in a 1:1 ratio to WDTE60N (*N* = 48) and placebo (*N* = 48) groups. Of these, six participants dropped out from the study; five of these were due to loss to follow‐up and one due to an accidental left leg injury that was determined to be unrelated to the study product after causality assessment. Consequently, 90 participants (WDTE60N, *n* = 45; placebo, *n* = 45) were considered for the final per‐protocol analysis (Figure 1).

The mean (SD) age and BMI of the participants were 31.82 (6.60) years and 26.76 (5.35) kg/m^2^, respectively. The demographic characteristics of the study population at baseline are summarized in Table 1. At baseline, the mean values for demographic characteristics and each endpoint were comparable between study groups (*p* > 0.05), indicating that the WDTE60N and placebo groups were comparable at the start of the study.

**TABLE 1:** Demographic details and baseline characteristics.

Parameters	WDTE60N (*N* = 48)	Placebo (*N* = 48)	Total (*N* = 96)	*p*
Age (years)	32.06 (6.71)	31.58 (6.55)	31.82 (6.60)	0.723
Body weight (kg)	62.00 (12.44)	63.21 (12.54)	62.60 (12.44)	0.636
Height (cm)	153.35 (5.51)	152.76 (5.66)	153.06 (5.56)	0.606
BMI (kg/m^2^)	26.39 (5.31)	27.13 (5.43)	26.76 (5.35)	0.501

*Note:* Data presented as mean (SD). *p*‐value derived from Student's unpaired *t*‐test.

Abbreviations: BMI, body mass index; SD, standard deviation; WDTE60N, water‐dispersible turmeric extract containing 60% natural curcuminoids.

### 3.2 Change in Pre‐Marked Blemish Areas on Facial Skin

**FIGURE 2:**
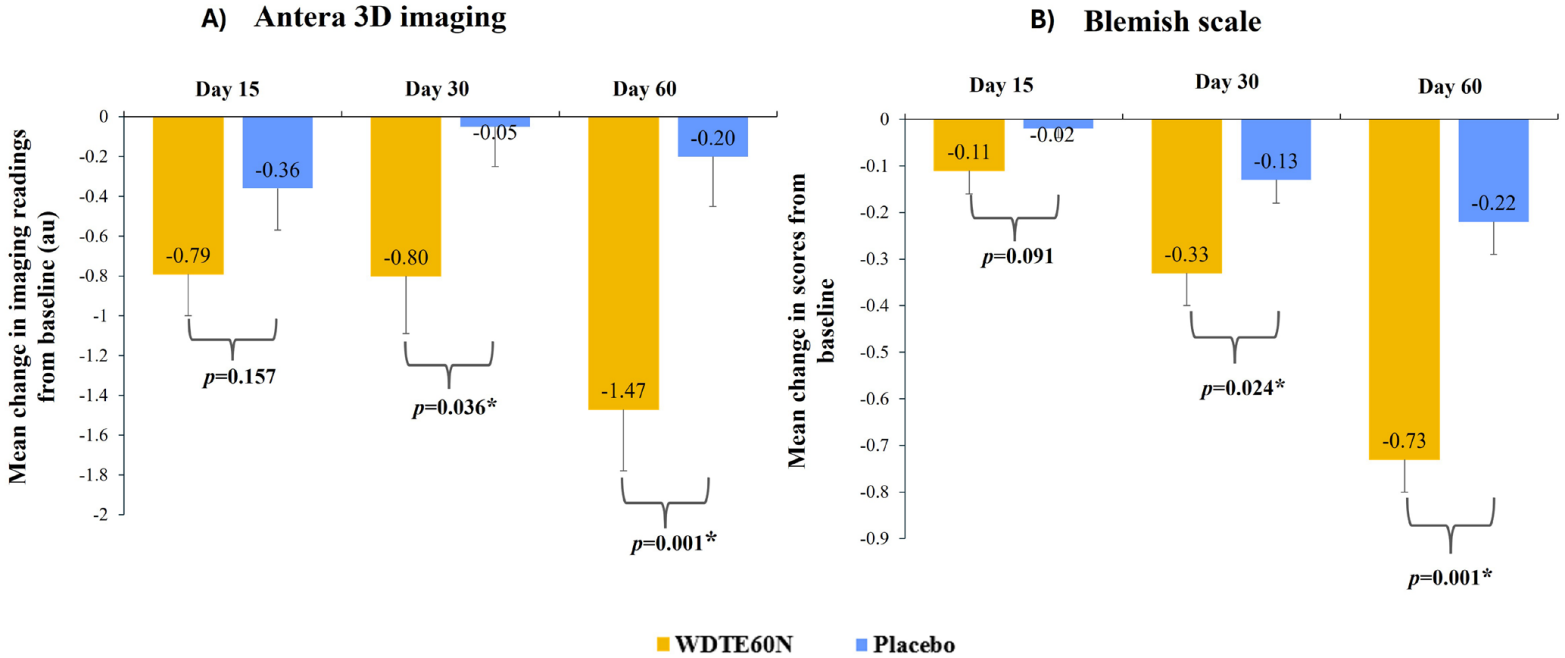
Comparison of change in pre‐marked blemish area between WDTE60N and placebo from baseline to day 60 using (A) Antera 3D imaging analysis; (B) Blemish scale analysis. Data presented as mean and SE (Error bars represent SE). * Represents statistically significant difference (*p*‐value derived from Student's unpaired *t*‐test). au, arbitrary units; SE, standard error; WDTE60N, water‐dispersible turmeric extract containing 60% natural curcuminoids.

**FIGURE 3:**
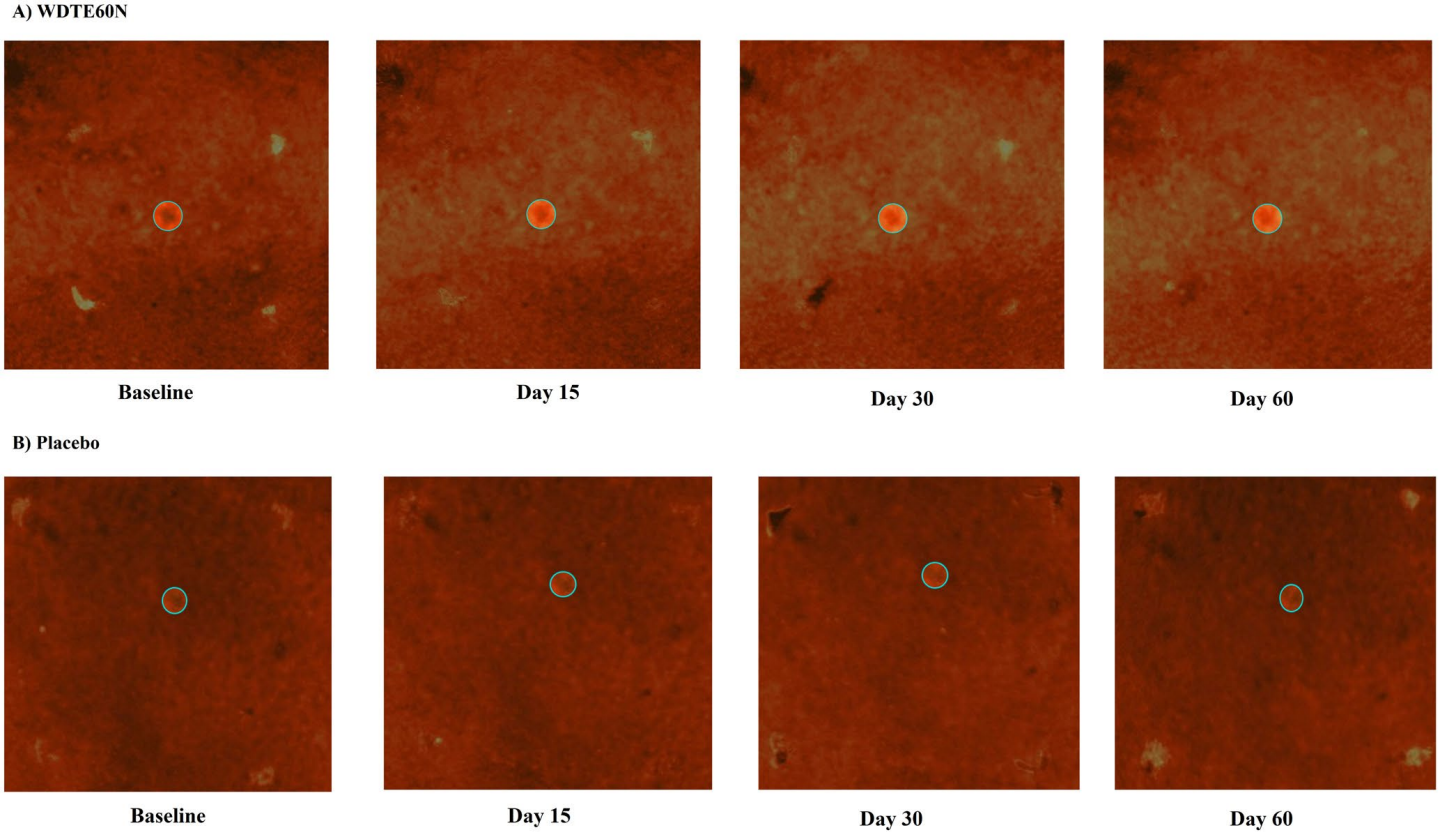
Representative Antera 3D images of blemish areas across study visits from (A) Participant administered with WDTE60N; (B) Participant administered with Placebo. WDTE60N, water‐dispersible turmeric extract containing 60% natural curcuminoids.

Antera 3D imaging analysis of pre‐marked blemishes showed that compared to the placebo group, the WDTE60N group showed statistically significantly higher mean change in Antera 3D imaging readings from day 30 onwards, which sustained till day 60 (Figure 2A). In the WDTE60N group, the mean (SD) Antera 3D imaging readings (au) showed a significant reduction from baseline (64.41 [7.06]) as early as day 15, which sustained till day 60 (day 15: 63.61 [7.32], *p* = 0.001; day 30: 63.61 [7.58], *p* = 0.008; day 60: 62.93 [7.38], *p* = 0.001). However, in the placebo group, no significant reduction in mean readings was observed from baseline to day 60. Representative images of the blemish areas of participants from WDTE60N and placebo groups for Antera 3D imaging analysis are as per Figure 3.

Clinical evaluation of pre‐marked blemishes using the blemish scale demonstrated a statistically significant reduction in mean (SD) score from baseline (3.00 [0.00]) on day 15, which continued through day 60 in the WDTE60N group (day 15: 2.89 [0.32], *p* = 0.025; day 30: 2.67 [0.48], *p* = 0.001; and day 60: 2.27 [0.50], *p* = 0.001) and from day 30 to day 60 in the placebo group (day 30: 2.87 [0.34], *p* = 0.013; day 60: 2.78 [0.47], *p* = 0.003), when compared with baseline (3.00 [0.00]). The mean changes from baseline to day 30 (*p* = 0.024) and day 60 (*p* = 0.001) were statistically significantly greater in the WDTE60N group than those observed in the placebo group (Figure 2B).

### 3.3 Change in Skin Hydration and TEWL

**FIGURE 4:**
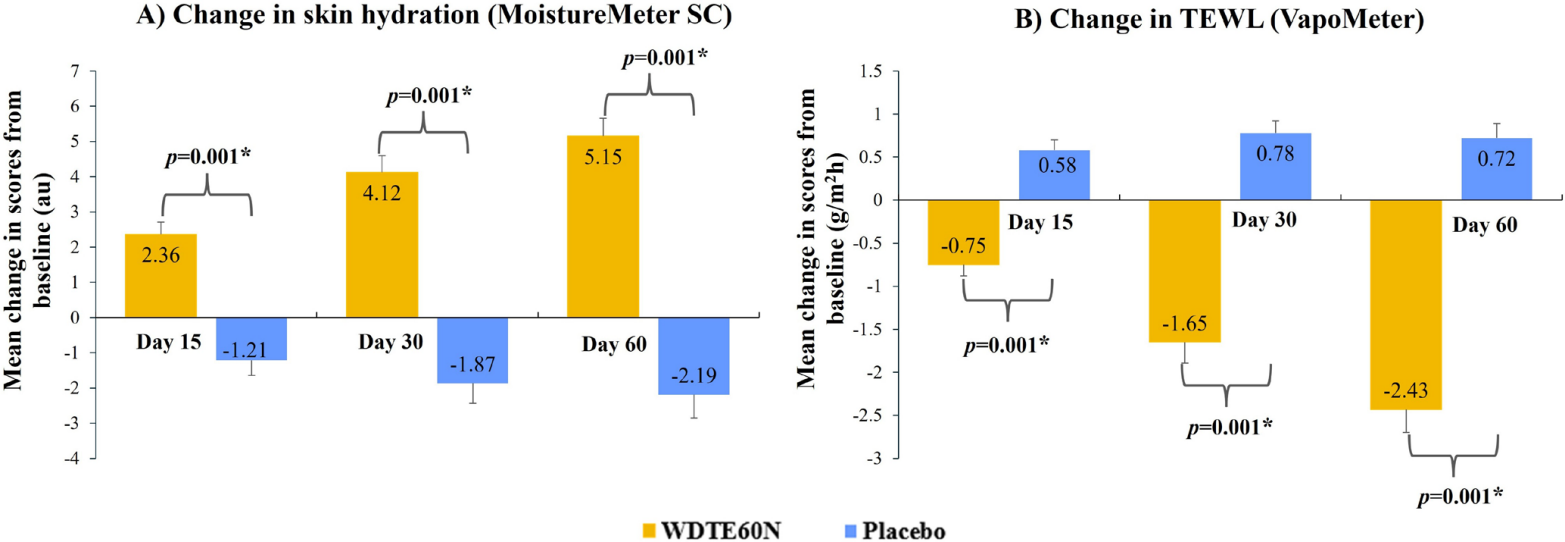
(A) Comparison of change in skin hydration between WDTE60N and placebo from baseline to day 60; (B) Comparison of change in TEWL between WDTE60N and placebo from baseline to day 60. Data presented as mean and SE (Error bars represent SE). * Represents statistically significant difference (*p*‐value derived from Student's unpaired *t*‐test). au, arbitrary units; SE, standard error; TEWL, transepidermal water loss; WDTE60N, water‐dispersible turmeric extract containing 60% natural curcuminoids.

Skin hydration evaluation using MoistureMeterSC demonstrated that in participants administered WDTE60N, a statistically significant increase in the mean (SD) skin hydration score (au) from baseline (19.31 [9.86]) was observed on day 15, which persisted through day 60 (day 15: 21.67 [9.23], *p* = 0.001; day 30: 23.43 [8.68], *p* = 0.001; day 60: 24.47 [8.97], *p* = 0.001); while in those administered placebo, a statistically significant decrease in mean hydration score (au) from baseline (19.46 [12.39]) was observed from day 15 to day 60 (day 15: 18.25 [10.47], *p* = 0.007; day 30: 17.59 [9.98], *p* = 0.001; day 60: 17.27 [9.94], *p* = 0.001). The comparison of mean change values from baseline on days 15, 30, and 60 between the groups revealed a statistically significant improvement in skin hydration among participants administered WDTE60N compared to those administered placebo (*p* = 0.001) (Figure 4A).

The skin barrier integrity assessed using VapoMeter demonstrated a statistically significant reduction in TEWL (g/m^2^h) from baseline (16.34 [4.63]) on day 15 to day 60 (day 15: 15.59 [4.10], *p* = 0.001; day 30: 14.69 [3.73], *p* = 0.001; day 60: 13.90 [3.64], *p* = 0.001) in participants from the WDTE60N group. In contrast, the participants from the placebo group showed a significant increase in the mean (SD) TEWL readings from baseline (15.10 [3.55]) to the end of the study (day 15: 15.68 [3.42], *p* = 0.001; day 30: 15.87 [3.42], *p* = 0.001; day 60: 15.82 [3.49], *p* = 0.001). The comparison of mean change from baseline on days 15, 30, and 60 between groups revealed a statistically significant decrease in TEWL readings in the WDTE60N group compared to placebo (Figure 4B).

### 3.4 Change in Skin Tone Evenness and Skin Gloss

Antera 3D imaging analysis conducted to assess change in facial skin tone evenness showed numerical reduction from baseline to end of the study in both the WDTE60N and placebo groups; however, the difference in mean reduction did not reach statistical significance between the WDTE60N and placebo groups (*p* > 0.05).

**FIGURE 5:**
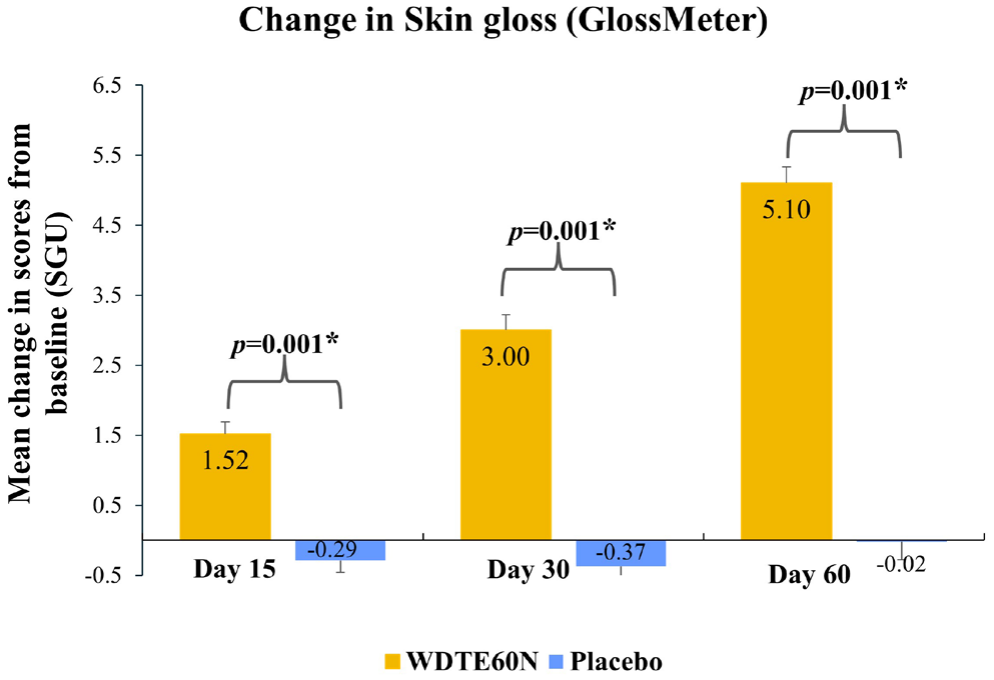
Comparison of change in skin gloss between WDTE60N and placebo from baseline to day 60. Data presented as mean and SE (Error bars represent SE). * Represents statistically significant difference (*p*‐value derived from Student's unpaired *t*‐test). SE, standard error; SGU, skin gloss units; WDTE60N, water‐dispersible turmeric extract containing 60% natural curcuminoids.

Evaluation of skin gloss using GlossMeter showed that the mean (SD) score (skin gloss units [SGU]) in participants from the WDTE60N group was statistically significantly increased from baseline (45.34 [5.40]) as early as day 15 (46.86 [5.42]) which was sustained through day 30 (48.34 [4.96]) and day 60 (50.44 [4.92]); while in the placebo group, within‐group differences in mean scores were comparable and showed a decrease from baseline on days 15 and 30 and then an increase on day 60. Mean changes in skin gloss from baseline to days 15, 30, and 60 were statistically significantly greater in the WDTE60N group than those observed in the placebo group (*p* = 0.001) (Figure 5).

### 3.5 Subjective Assessment of Efficacy of Study Treatments in Improving Facial Skin Parameters

A self‐assessment efficacy questionnaire was used to evaluate participants' perception about the efficacy of study treatments on various skin properties. A mean score greater than 3.5 was considered a positive indication of improvement in the efficacy parameter. Compared to the placebo group, participants in the WDTE60N group reported markedly higher improvement in each efficacy parameter from baseline to day 60 (*p* < 0.05). Participants reported the effectiveness of WDTE60N in imparting healthy glowing skin, smoothening the skin tone, and reducing the appearance of dark spots from day 15 onwards, while from day 30, they also reported its efficacy in maintaining their skin soft, smooth, moisturized, and blemish‐free, which continued through day 60 (Table 2).

**TABLE 2:** Subjective assessment using self‐assessment questionnaire for efficacy.

Self‐assessment questions	WDTE60N				Placebo			
	Baseline	Day 15	Day 30	Day 60	Baseline	Day 15	Day 30	Day 60
Makes my skin feel softer and smoother	2.98 (0.87)	3.56 (0.66)^$^, *	3.96 (0.42)^$^, *	4.16 (0.47)^$^, *	3.16 (0.88)	3.40 (0.72)	3.51 (0.76)^$^, *	3.44 (0.84)
Mean change from baseline	—	0.58	0.98^#^, *	1.18^#^, *	—	0.24	0.36	0.29
Keeps my skin moisturized	2.98 (0.84)	3.62 (0.61)^$^, *	3.87 (0.50)^$^, *	4.02 (0.50)^$^, *	3.04 (0.77)	3.40 (0.75)^$^, *	3.24 (0.77)	3.18 (0.83)
Mean change from baseline	—	0.64	0.89^#^, *	1.04^#^, *	—	0.36	0.20	0.13
Gives my skin a healthy glow	2.67 (0.83)	3.47 (0.76)^$^, *	3.96 (0.47)^$^, *	4.16 (0.56)^$^, *	2.62 (0.75)	3.00 (0.83)^$^, *	3.09 (0.85)^$^, *	3.18 (0.91)^$^, *
Mean change from baseline	—	0.80^#^, *	1.29^#^, *	1.49^#^, *	—	0.38	0.47	0.56
Evens out my skin tone	2.40 (0.65)	3.31 (0.76)^$^, *	3.76 (0.61)^$^, *	3.93 (0.54)^$^, *	2.29 (0.63)	2.76 (0.86)^$^, *	2.82 (0.86)^$^, *	2.87 (0.87)^$^, *
Mean change from baseline	—	0.91^#^, *	1.36^#^, *	1.53^#^, *	—	0.47	0.53	0.58
Reduces the appearance of dark spots	2.04 (0.21)	3.31 (0.79)^$^, *	3.80 (0.63)^$^, *	4.18 (0.65)^$^, *	2.02 (0.15)	2.71 (0.79)^$^, *	2.84 (0.85)^$^, *	2.89 (0.83)^$^, *
Mean change from baseline	—	1.27^#^, *	1.76^#^, *	2.13^#^, *	—	0.69	0.82	0.87
Keeps my skin blemish free	2.04 (0.21)	2.58 (0.66)^$^, *	3.13 (0.73)^$^, *	3.91 (0.51)^$^, *	2.00 (0.00)	2.42 (0.66)^$^, *	2.53 (0.73)^$^, *	2.69 (0.76)^$^, *
Mean change from baseline	—	0.53	1.09^#^, *	1.87^#^, *	—	0.42	0.53	0.69

*Note:* Data presented as mean (SD), unless otherwise specified. Scale used: 1 = Strongly Disagree; 2 = Disagree; 3 = Neither agree nor disagree; 4 = Agree; 5 = Strongly Agree.

* Represents *p‐*value < 0.05, which indicates a statistically significant difference.

^#^ Shows comparison of mean scores between baseline and each follow‐up time point (*p*‐value derived from Wilcoxon Signed‐Rank test).

^$^ Shows comparison of the mean change from baseline to all follow‐up timepoints between WDTE60N and placebo (*p‐*value derived from Mann Whitney *U* test).

### 3.6 Subjective Assessment of In‐Use Tolerance

A total of two participants in the WDTE60N group noted bloating (*n* = 1) and diarrhea (*n* = 1), which were mild in intensity. Whereas six participants in the placebo group noted mild acid reflux (*n* = 1), burping (*n* = 1), and mild constipation (*n* = 1); mild discomfort in the upper abdomen (*n* = 1); mild bloating (*n* = 1), and mild diarrhea (*n* = 1).

### 3.7 Safety Assessment

During the study, two participants from the placebo group reported moderate AEs (accidental left leg injury: *n* = 1, who later discontinued from the study; body ache and joint pain: *n* = 1). On day 60, post‐study lab assessments revealed that one participant from the WDTE60N group had abnormal thyroid‐stimulating hormone levels and was advised to consult a family physician for further management. The reported AEs in both study groups were medically managed, assessed, and concluded to be unrelated to the study treatments and were resolved without sequelae.

## 4 Discussion

In the present study, the efficacy of WDTE60N, administered in the dose of a 250 mg capsule once daily for 60 days, was evaluated for its impact on facial skin parameters using both objective and subjective assessments. Observations from objective assessments demonstrated that compared to placebo, WDTE60N significantly reduced facial blemishes from day 30 onward, which continued till day 60. Additionally, it markedly enhanced skin hydration and skin gloss while reducing TEWL from as early as day 15, with these benefits sustained through day 60. In subjective assessments, participants from the WDTE60N group reported noticeable improvements in skin softness, smoothness, and hydration, and achieving blemish‐free skin from day 30 onward. Additionally, they reported enhanced skin glow, reduced dark spots, and an evenness of the skin tone from as early as day 15, with effects persisting throughout the study duration. Overall, these observations indicate that administration of WDTE60N at a single daily dose for 2 months was effective in improving facial skin health among healthy adult women.

In the present study, the effect of WDTE60N versus (vs.) placebo administration on facial blemishes using Antera 3D imaging analysis revealed a significant reduction in blemish readings in the WDTE60N group compared to placebo, starting from day 30 and persisting through day 60, indicating a sustained reduction in facial blemish. Notably, by day 60, the decrease in facial blemish in the WDTE60N group was approximately 7.35 times greater than in the placebo group (percentage reduction from baseline: 2.28% vs. 0.31%). These instrument‐based findings were further supported by clinical assessment using the blemish scale. In the WDTE60N group, a significant reduction in blemish scale scores was observed from day 30, which continued through day 60, compared to placebo. At baseline, both study groups had a mean blemish score of 3, indicating moderate dark blemishes. By day 60, the blemish score was reduced to 2.27 in the WDTE60N group and 2.78 in the placebo group. These findings suggest that WDTE60N administration effectively reduced facial blemishes, reducing their severity from moderate to mild. In the present study, we identified a single facial blemish resulting from post‐inflammatory hyperpigmentation, sunspots, age spots, or acne marks. Typically, post‐inflammatory hyperpigmentation arises from excess and uneven dispersion of melanin in the epidermis or dermis, triggered by various skin insults, such as acne, trauma, or dermatologic procedures, which further release inflammatory cytokines, prostaglandins, and reactive oxygen species, stimulating melanocytes to overproduce melanin. The excess melanin pigment is then transferred to surrounding keratinocytes in the epidermis and, in some cases, leaks into the dermis, ultimately forming the blemish [22]. The decrement in the blemish intensity after WDTE60N administration in the present study might be attributed to the antioxidant and anti‐tyrosinase properties of curcuminoids. Additionally, turmeric may help reduce melanogenesis and pigmentation by inhibiting melanocyte‐stimulating hormone [23, 24]. This is substantiated by a study showing that turmeric extract suppresses ultraviolet A (UVA)‐induced melanogenesis and oxidative stress in a dose‐dependent manner, suggesting a connection between its antioxidant and anti‐tyrosinase properties [25]. Another study that examined the effects of turmeric tablets and turmeric‐containing herbal combination tablets (4000 mg) demonstrated approximately 20% reduction in facial pigmentation and wrinkle severity in the turmeric group and approximately 1% in the combination group, with no decrease in the placebo group, though the results were not statistically significant [13].

Optimal hydration, an even tone, absence of hyperpigmentation, and a natural glow are key indicators of healthy skin. Among these, skin hydration plays a pivotal role in supporting normal skin function and overall skin health [26]. In the present study, assessment using the MoistureMeterSC revealed a significant increase in skin hydration from baseline to day 60 in participants receiving WDTE60N, whereas a decline in skin hydration was observed in the placebo group over the same period. Notably, this skin‐hydrating effect of WDTE60N was evident from as early as day 15 (percentage change from baseline: 12.22% vs. −6.22%), which continued to improve through day 30 (21.34% vs. −9.61%) and day 60 (26.67% vs. −11.25%). Administration of the test product transformed the facial skin from dry (< 20) to the normal skin (range of 20–40) from day 15 onwards. The subjective evaluation also corroborated with the above results, showing noticeable improvements in skin hydration from day 30 onwards. Thus, these findings strongly support the efficacy of WDTE60N in enhancing skin hydration. As hydration plays a vital role in maintaining skin suppleness and elasticity, WDTE60N may aid in enhancing the suppleness of facial skin [27].

The present study assessed the effect of IPs on skin barrier integrity by measuring TEWL, a widely accepted objective non‐invasive method, using the VapoMeter [28, 29, 30]. WDTE60N administration led to a significant reduction in TEWL from baseline, with decreases of 4.59% on day 15, 10.10% on day 30, and 14.87% on day 60, indicating a sustained improvement in skin barrier integrity over the study duration. However, the placebo group exhibited an opposite trend, with TEWL increasing from baseline to day 60, suggesting deterioration of skin barrier integrity. These findings are supported by Vaughn et al.'s study, which reported a significant reduction in TEWL after 4 weeks of twice‐daily use of turmeric‐containing herbal combination tablets (500 mg proprietary blend of herbs), and a trend toward decreased TEWL, although non‐significant, in the turmeric tablets (500 mg turmeric root) group as compared to placebo [13]. The observations from the present study further reinforce the role of WDTE60N in enhancing facial skin hydration by strengthening skin barrier integrity and moisture retention, probably contributed through the anti‐inflammatory and antioxidant properties of curcuminoids [31].

Skin gloss is another key skin metric evaluated, and WDTE60N administration resulted in a significant improvement in this parameter from baseline. Objective assessment demonstrated the efficacy of WDTE60N compared to placebo in enhancing skin radiance, with improvements observed as early as day 15 (percentage change from baseline: 3.35% vs. −0.60%), which continued to further improve through day 30 (percentage change from baseline: 6.62% vs. −0.77%) and day 60 (percentage change from baseline: 11.25% vs. −0.04%). Similarly, participants from the WDTE60N group subjectively reported a noticeable improvement in skin glow from day 15 onwards. Taken together, WDTE60N supplementation proved to be both effective and faster in enhancing skin radiance compared to the placebo. The dearth of studies evaluating the effect of oral or topical turmeric extract on skin gloss limited the comparison of these findings with existing literature.

Subjective assessment scores of other skin attributes revealed that participants perceived WDTE60N as effective in promoting a more even skin tone (from day 15 onwards), in reducing the dark spots appearance (from day 15 onwards), and in making skin softer and smoother, and blemish‐free (from day 30 onwards). Safety evaluations showed that AEs reported during the study period were of moderate intensity and were not related to the study products, thus confirming that WDTE60N was well‐tolerated by the participants.

### 4.1 Strengths and Limitations of Study

Although turmeric has been well‐established as a skin care ingredient since ancient times, and a growing number of clinical studies have demonstrated its efficacy—either as a topical application or an ingested extract, alone or in combination with other therapies—across a wide range of skin disorders, research on its effectiveness and safety as a dietary supplement for enhancing facial skin health in a healthy population remains scarce. Based on the extensive literature review, this is the pioneering study evaluating the efficacy and safety of the oral supplementation of a low‐dose water‐dispersible turmeric extract over two months in managing a comprehensive range of skin health parameters in healthy young to middle‐aged (18–40 years) women. Notably, despite participants enrolled in this study being generally healthy and free from overt skin diseases—conditions where changes in pigmentation are typically more difficult to detect—a reduction in blemish intensity was evident as early as day 30, thus underscoring the promising potential of WDTE60N in reducing facial skin blemishes at a faster rate.

This study highlights the efficacy of WDTE60N as a unique beauty‐from‐within solution, with efficacy achieved in a single daily dose for 2 months. Earlier studies have also confirmed its musculoskeletal health benefits pertaining to its anti‐inflammatory effect and supported by significantly reduced inflammatory cytokine levels in the clinical setup [32, 33]. It is probably due to its potent anti‐inflammatory mechanism combined with its antioxidant and collagen‐promoting effects that WDTE60N has shown its efficacy in remarkably enhancing facial skin health [10, 34]. The dose selected for this study is based on two pharmacokinetic studies of WDTE60N wherein the optimum bioavailability of the product at a single dose of 250 mg has been shown to be comparable with 1500 mg of standard 95% turmeric extract [14, 15]. Interestingly, the product demonstrated its efficacy at the same dosage in a comparatively novel health application area, for which it has been very commonly used traditionally for centuries.

However, a few limitations of this study must be acknowledged. The exclusion of male participants restricts the generalizability of WDTE60N's effects on facial skin improvement among men. Additionally, as optimal skin health is important across all age groups, future research should explore its effects on skin health in adolescents and the elderly populations to gain a more comprehensive understanding. Another limitation is the short study duration, which prevents assessment of the long‐term effects of oral turmeric extract supplementation on skin health. Future studies with longer follow‐up periods and a more diverse population will be essential to further validate the benefits of WDTE60N and provide a more holistic understanding of its role in improving facial skin health.

## 5 Conclusions

Overall findings from objective and subjective assessments indicate that the two‐month supplementation with a WDTE60N 250 mg capsule once a day was safe and effective in improving facial skin health. Particularly, facial blemish reduction was observed from day 30, while improvement in skin hydration, skin barrier integrity, and gloss was evident from day 15 and sustained through the end of the study. These benefits may be attributed to the antioxidant, anti‐tyrosinase, and anti‐inflammatory effects of curcuminoids present in WDTE60N, which help mitigate oxidative stress, excessive melanin production, and inflammation—factors that contribute to the above‐mentioned skin concerns. Collectively, these findings highlight the role of WDTE60N in improving key attributes of facial skin health, opening new possibilities in women's skincare as a cosmeceutical ingredient supporting beauty from within while also providing other health benefits.

## Author Contributions

**Shefali Thanawala:** contributed to conceptualization, data curation, formal analysis, project administration, supervision, writing – original draft and critical revision of the manuscript. **Rajat Shah:** contributed to conceptualization, funding acquisition, providing resources, visualization, and reviewing and editing of manuscript. **Krishnaraju Venkata Alluri:** contributed to data curation, formal analysis, project administration, supervision, reviewing and editing of manuscript. **Kiran Bhupathiraju:** contributed to funding acquisition, providing resources, and reviewing and editing of the manuscript. **Anjali Salvi:** contributed to data curation, formal analysis, investigations and reviewing and editing of the manuscript.

## Ethics Statement

The study protocol was approved by CLAIMS Independent Ethics Committee (Re‐Registration number: ECR/245/Indt/MH/2015/RR‐22; Approval Code: CL/172/0124/STU; Approval Date: 31/05/2024). The study protocol adhered to the ethical guidelines set forth by the Declaration of Helsinki for research involving human participants. The study was conducted in compliance with the ICMR guidelines—National Ethical Guidelines for Biomedical and Health Research Involving Human Participants, 2017, International Conference on Harmonization‐Good Clinical Practices (ICH‐GCP) guidelines E6 (R2), NDCT RULES 2019, Declaration of Helsinki (Brazil, October 2013), and other applicable regulatory requirements.

## Consent

Written informed consent was provided by each participant before the initiation of the study.

## Conflicts of Interest

Ms. Rajat Shah and Dr. Shefali Thanawala are employees of Nutriventia Private Limited. Ms. Rajat Shah also has ownership interests. Dr. Krishnaraju Venkata Alluri and Mr. Kiran Bhupathiraju are employees of Laila Nutraceuticals, India. Dr. Anjali Salvi was the co‐investigator in this study. The authors do not have any other conflicts of interest to declare.

## Data Availability

The data that support the findings of this study are available from the corresponding author upon reasonable request.
